# Efficient Magnetic Vortex Acceleration by femtosecond laser interaction with long living optically shaped gas targets in the near critical density plasma regime

**DOI:** 10.1038/s41598-024-54475-1

**Published:** 2024-02-28

**Authors:** I. Tazes, S. Passalidis, E. Kaselouris, D. Mancelli, C. Karvounis, A. Skoulakis, I. Fitilis, M. Bakarezos, N. A. Papadogiannis, V. Dimitriou, M. Tatarakis

**Affiliations:** 1 Institute of Plasma Physics and Lasers-IPPL, University Research and Innovation Centre, Hellenic Mediterranean University, 74100 Rethymno, Greece; 2 Department of Electronic Engineering, Hellenic Mediterranean University, 73133 Chania, Greece; 3 CEA, DAM, DIF, 91297 Arpajon, France; 4 Université Paris-Saclay, CEA, LMCE, 91680 Bruyères-le-Châtel, France; 5 Physical Acoustics and Optoacoustics Laboratory, Department of Music Technology and Acoustics, Hellenic Mediterranean University, 74100 Rethymno, Greece

**Keywords:** Computational science, Laser-produced plasmas

## Abstract

We introduce a novel, gaseous target optical shaping laser set-up, capable to generate short scale length, near-critical target profiles via generated colliding blast waves. These profiles are capable to maintain their compressed density for several nanoseconds, being therefore ideal for laser-plasma particle acceleration experiments in the near critical density plasma regime. Our proposed method overcomes the laser-target synchronization limitations and delivers energetic protons, during the temporal evolution of the optically shaped profile, in a time window of approximately 2.5 ns. The optical shaping of the gas-jet profiles is optimised by MagnetoHydroDynamic simulations. 3D Particle-In-Cell models, adopting the spatiotemporal profile, simulate the 45 TW femtosecond laser plasma interaction to demonstrate the feasibility of the proposed proton acceleration set-up. The optical shaping of gas-jets is performed by multiple, nanosecond laser pulse generated blastwaves. This process results in steep gradient, short scale length plasma profiles, in the near critical density regime allowing operation at high repetition rates. Notably, the Magnetic Vortex Acceleration mechanism exhibits high efficiency in coupling the laser energy into the plasma in the optically shaped targets, resulting to collimated proton beams of energies up to 14 MeV.

## Introduction

Laser-induced particle acceleration has attracted extensive interest due to basic physics curiosity but also due to its numerous potential applications. Such important applications include among others, laser driven Inertial Fusion Energy (IFE) associated with the proton driven fast ignition^1–3^, proton-boron fusion studies^4–6^ as well as hadron therapy studies^7–11^.

The recent achievement of laser driven fusion ignition at the National Ignition Facility (NIF) sets a historic milestone in fusion energy research^12^. The breakeven at NIF produced a total of 3.15 MJ of fusion energy with 2.05 MJ of laser input energy, demonstrating that the laser driven IFE concept works, efforts in the coming decades will focus on developing a path to exploring alternative more efficient laser driven IFE schemes to address aspects related to high gain fusion energy production, including high repetition rate laser technology as well as efficient target manufacturing. In the case of the ion fast ignition scheme, high repetition rate ion sources are technically limited by the use of solid targets, even if sophisticated complex layouts are being developed.

Protons of hundreds of MeV have been produced in the Target Normal Sheath Acceleration (TNSA) and Radiation Pressure Acceleration (RPA) regimes^13–16^. The irradiation upon TNSA and RPA destroys the solid targets and therefore repositioning is demanded after a single or a series of laser shots, thus limiting the ability of high repetition rate. On the other hand, gaseous targets are the most promising alternative supporting the high repetition rates needed and generating almost debris-free proton sources^17,18^. But their use as proton sources is still challenging since extremely high densities are demanded. In this regime, the dominant acceleration mechanisms are the Magnetic Vortex Acceleration (MVA)^19–27^, the Collisionless Shock Acceleration (CSA)^28–30^ and the Coulomb Explosion (CE) from atomic clusters^31,32^. Protons and ions have been experimentally accelerated in the past in gaseous targets, up to 20 MeV per nucleon by high energy, ns pulse duration, CO_2_ lasers at “single shot” experiments^33,34^. These relatively low intensity lasers may access their near-critical density regime at lower densities due to their longer wavelengths (critical density n_cr_ ≈ 10^19^ cm^−3^, λ = 10 μm). State-of-the-art simulations predict hundreds of MeV up to a GeV of protons accelerated in near-critical density plasmas via MVA, by intense Ti:Sa fs laser pulses^19–26^. To the best of our knowledge, experimental verification of ion acceleration in the MVA regime with typical fs laser wavelengths does not exist up to date, since extremely dense and sharp plasma profiles are necessary as implied by the simulations^19–26^.

In our previous works we have studied the feasibility of proton acceleration by the interaction of super-intense fs laser pulses with high pressure, optically shaped gas-jet profiles studied and optimized by MagnetoHydroDynamic (MHD) simulations^35,36^. Therein, Sedov type colliding blastwaves (BWs) were computationally generated within the gas-jet, having high pressure and hundreds of microns scale length^35–39^. The shock fronts of those counterpropagating BWs are colliding close to the peak density of the gas-jet, briefly compressing its initial density several times. The compressed structures developed are characterised by a typical thickness of just a few tens of microns, while in alternative geometrical set-ups the elimination of low density long scale length pedestals were indicated. Density variations within the target profile volume may adversely affect the focusing of the accelerating laser pulse. Typically, the compression of a gas target is sustained just for a few hundreds of picoseconds^36,37^ and this is the major drawback for the experimental realisation due to serious difficulties in reliable main laser pulse-target synchronization.

Here, we propose for the first time a method able to generate cylindrical compressed target profiles sustaining their density profile structure for several nanoseconds. These profiles are characterized by a long and controllable lifetime able to provide near critical densities allowing for an achievable and efficient synchronisation of the femtosecond laser pulse with the compressed gaseous target. In particular, we focus on the study of the interaction of the Zeus laser system with the aforementioned target profiles. Zeus laser is hosted at the Institute of Plasma Physics and Lasers (IPPL)^40^ of the Hellenic Mediterranean University (HMU) at Rethymno, Greece, delivering 45 TW, 23 fs, 1.1 J Ti:Sa pulses^36,40–43^. This interaction yields energetic protons, throughout the temporal evolution of the optically shaped high density gas in a compression time window of several nanoseconds.

## Magnetic Vortex Acceleration theoretical description

When the intense fs laser pulse propagates in a gaseous target, it penetrates the near critical density regions and ponderomotively expel electrons, thus creating a low electron density channel into the plasma. Therefore, a positively charged region behind the pulse is formed, since the ion's response time is slow. The laser pulse accelerates the electrons on its wake, as it propagates through the channel, in a thin electron filament resulting in a strong electric current, while the cold electrons of the channel walls propagate backwards generating a return current. As a result, a strong azimuthal quasistatic magnetic field is produced which remains confined in the channel. The propagation of the pulse through the channel is approximated by the propagation of an electromagnetic (EM) field into a waveguide. As the laser pulse penetrates the dense region and escapes from the rear of the target, the confined magnetic field starts to expand in the transverse direction generating a strong longitudinal electric field, which accelerates the ions. The accelerated ion filament is further pinched by the magnetic vortex. MVA demands the efficient conversion of the laser energy to the acceleration of fast electrons through the channel and along the direction of the propagating laser. The optimal ion acceleration can be estimated if the laser pulse energy inside the waveguide becomes equal to the energy of the heated electrons. The equations describing the optimum acceleration conditions are derived under the assumption that the total laser energy is transferred to the electrons in the plasma channel^19–22^. Since for a given ion density an optimal target width can be calculated, the optimization of MVA requires the coupling of the target density and the length of the generated channel inside the target. The optimal coupling condition between the electron density and the plasma channel length is described by^20–22^:

1 $$\frac{{n}_{e}}{{n}_{cr}}=\sqrt{2}K{\left(\frac{P}{{P}_{c}}\right)}^{1/2}{\left(\frac{{L}_{p}}{{L}_{ch}}\right)}^{3/2}$$

where, *n*_*e*_ is the electron density, *n*_*cr*_ is the critical electron density, *Κ* is a geometrical factor with the value of 1/10 in 2D and 1/13.5 in 3D domains, *P* is the power of the laser pulse, *P*_*c*_ = 17.3 GW is the power of the relativistic self-focusing^44^, *L*_*p*_ is the length of the laser pulse and *L*_*ch*_ is the length of the generated plasma channel. Given the Zeus laser pulses characteristics having a wavelength *λ* = 805 nm, *n*_*cr*_ = 1.74 10^21^ electrons/cm^3^_,_ *P* = 45 TW, *L*_*p*_ $\approx$ 11.7 μm for the 23 fs pulse duration and *n*_*e*_ $\approx$ 0.5 *n*_*cr*_^36^, Eq. (1) results to the optimal length of the laser generated plasma channel being *L*_*ch*_ $\approx$ 40 μm.

The strength of the magnetic field generated inside the channel, at a radius equal to the electron beam radius, by the accelerated electrons is given by^19^:

2 $${B}_{ch}=2\pi e{n}_{e}{R}_{ch}{\gamma }_{e}^{2}$$

where *e* is the electron charge, *γ*_*e*_ is the electron relativistic Lorentz factor and *R*_*ch*_ is the radius of the channel^16–18^. The longitudinal electric field, accelerating the ions at the rear of the target, is generated by the expanding magnetic field with strength in the order of the *B*_*ch*_*.* An upper limit for the accelerated particles cut-off energies can be estimated by^24^:

3 $${E}_{i}={m}_{e}{c}^{2}2{\pi }^{2}{Z}_{i}\frac{{n}_{e}}{{n}_{cr}}{\left(\frac{{R}_{ch}}{\lambda }\right)}^{4}$$

where *m*_*e*_ is the electron mass and Z_i_ is the ionization degree.

## Results and discussion

**Figure 1:**
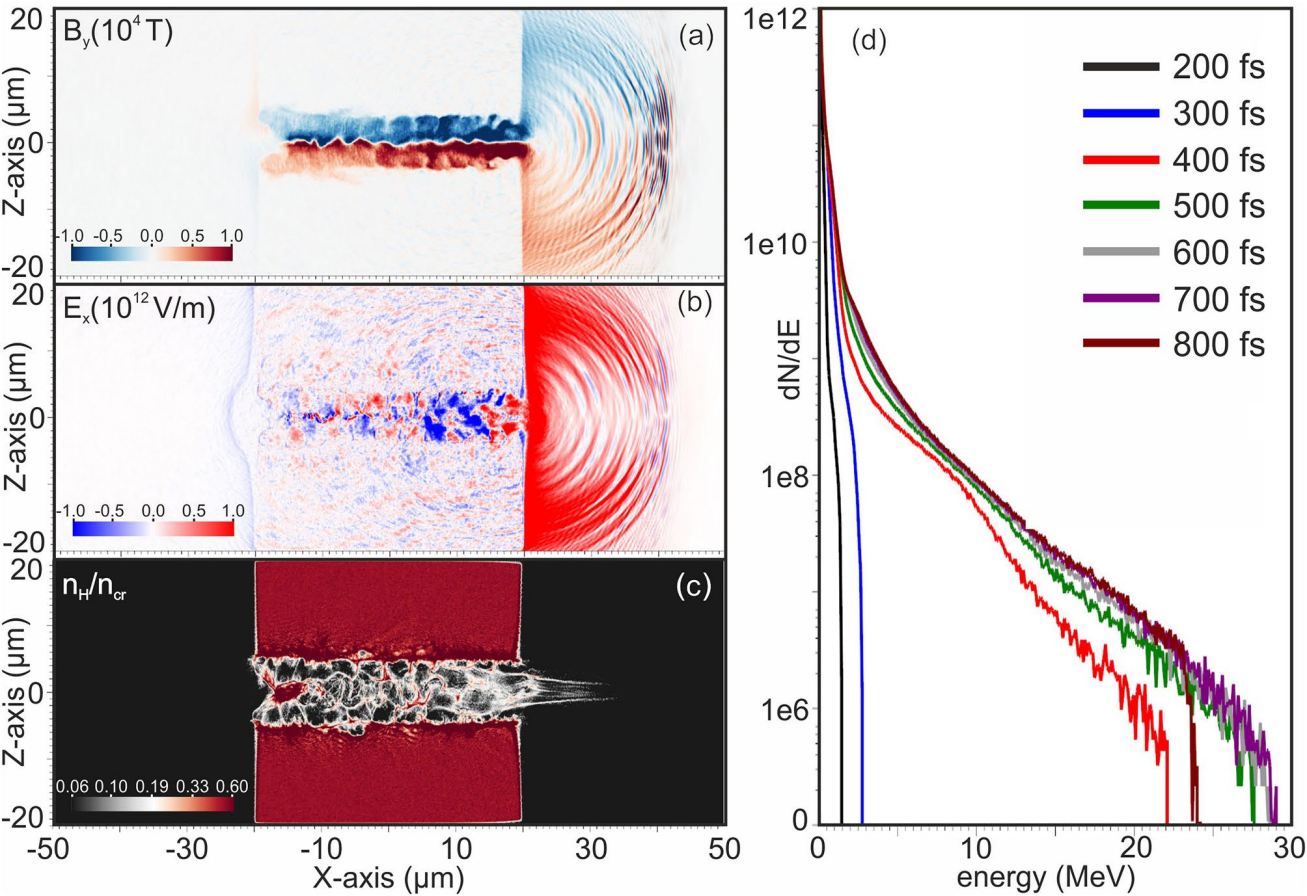
PIC simulation results of the interaction of the Zeus 45 TW, fs laser pulse with the top-hat near critical density target. (**a**) Azimuthal magnetic field B_y_, (**b**) accelerating, longitudinal electric field E_x_, (**c**) logarithmic plot of the proton density expressed as n_H_/n_cr,_ (**d**) temporal evolution of the accelerated protons energy spectra.

We aim to accurately determine the MVA mechanism and the resulting proton energies, derived by the proposed optical shaping scheme. A commonly used PIC model was used to simulate the 45 TW Zeus femtosecond laser pulse, initially interacting with a top-hat density distributed gas target, as a reference simulation^20–22^. The computational domain, the main laser pulse characteristics and the transient parameters implied, were based on the experimental set-ups developed in IPPL and were also directly adopted to simulate the same interaction with the proposed BWs optimally shaped gas target profiles^36^. Figure 1a–c show the computational domain size 100 × 40 × 40 μm. The laser pulse propagates along the X axis where the top-hat distribution of the density initiates at -20 μm and is extended up to + 20 μm, according to Eq. (1). At the 300^th^ fs, the main laser pulse penetrates the plasma-vacuum interface at the front of the target (X = 20 μm) and the azimuthal vortex magnetic field starts to transversely expand. When the main laser pulse penetrates the rear plasma vacuum interface, the magnetic field’s peak value exceeds the 6 × 10^4^ T. At the 350^th^ fs the magnetic field is fully developed having a maximum value of 2 × 10^4^ T, and the induced longitudinal accelerating electric field *E*_*x*_ has a maximum value 5 × 10^12^ V/m, as shown in Fig. 1a,b, respectively. The diverging accelerated proton bunch propagating inside the vacuum behind the top-hat target, is presented in Fig. 1c at the 600th fs. The temporal evolution of the accelerated protons kinetic energy in Fig. 1d, demonstrates that protons have acquired their maximum energy, being higher than 28 MeV, before the 500 fs, in agreement with the theoretical results of Eqs. (2), (3) ^20–22^.

**Figure 2:**
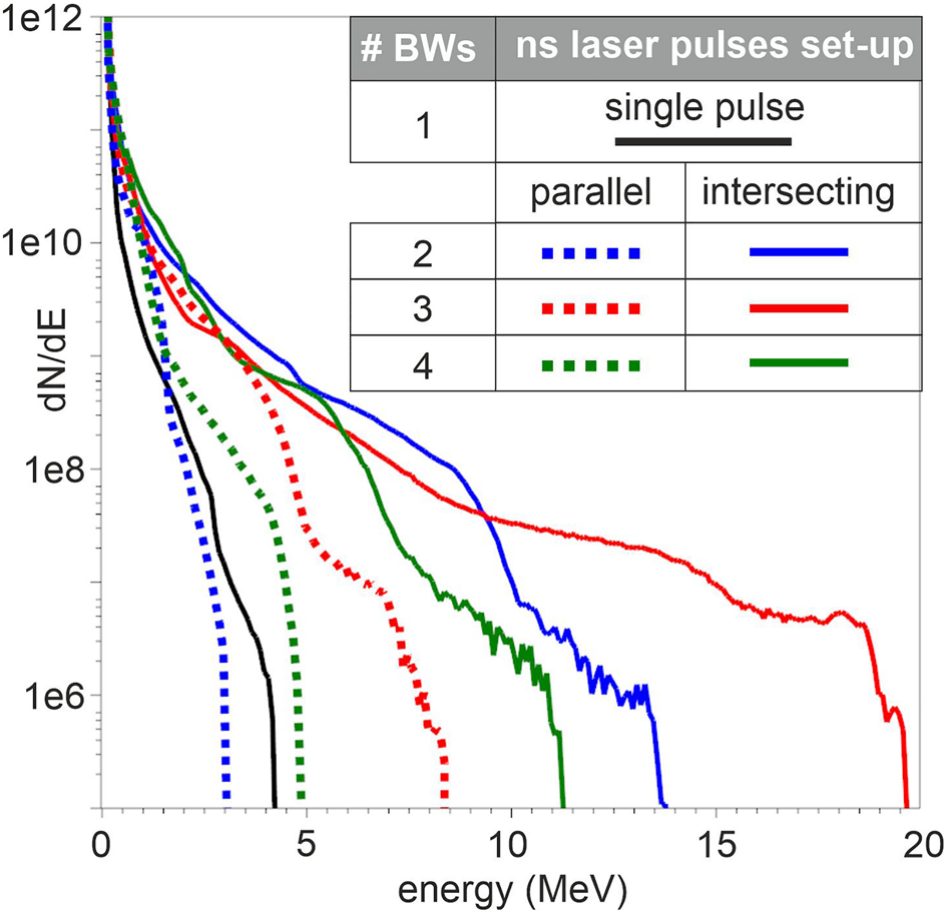
PIC simulation results energy spectra of the optically shaped gas target profiles. The black solid line refers to the density profile shaped from a single ns laser pulse. The geometrical set-ups, of the parallel and the intersecting ns laser pulses, are represented by dashed and solid lines correspondingly.

Based on the initial and boundary conditions described, we further studied the MVA using the optically shaped gas target profiles generated in by MHD simulations. Figure 2 shows PIC simulated energy spectra of protons accelerated by the Zeus laser pulse interacting with the optically shaped gas target. The target is shaped by colliding BWs produced by one up to four laser pulses with 5 ns duration, in parallel and intersecting geometries^36^. The solid black line corresponds to the irradiation of the shock front of a single BW while the others denote the irradiation of the multiple BWs at the time of their shock front collisions when maximum compression is achieved.

**Figure 3:**
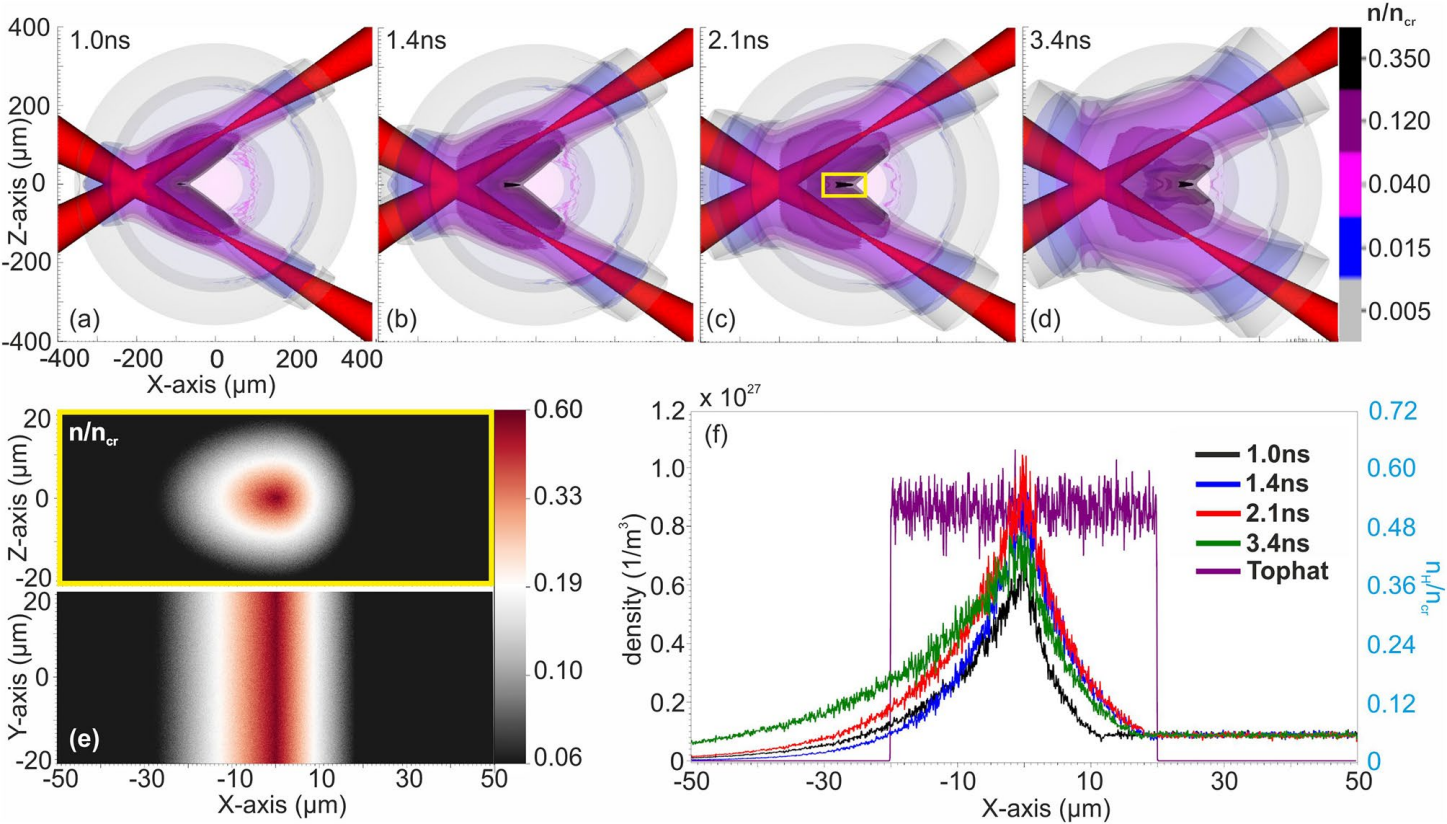
Optical shaping of the gas-jet profile generated by the two intersecting ns laser pulses set-up. (**a**–**d**) MHD simulation results of the density temporal evolution for 1.0, 1.4, 2.1 and 3.4 ns. The colormap is saturated at 0.35 n_cr_ while the maximum value of the density is 0.55 n_cr_. (**e**) The initial density distribution of the cylindrically shaped compressed profile on the XZ (top) and the XY (bottom) planes of the PIC models at 2.1 ns. (**f**) Lineouts of the initial density profiles at 1.0, 1.4, 2.1 and 3.4 ns plotted along with the top-hat reference density profile.

The results presented in Fig. 2, highlight that higher proton energies were achieved when the ns laser pulses were intersected to generate the colliding BWs. In the intersecting ns laser pulses set-ups, gas target profiles with high densities and steep density gradients were generated, in contrast to the parallel pulse layouts where long scale length pre-plasma pedestals were present, thus decreasing the efficiency of proton acceleration due to the laser pulse filamentation^45^. The maximum cut off energy was achieved when the density profile shaped by the three intersecting ns laser pulses generated BWs was used. A density of ~ 0.75 *n*_*cr*_ was reached during its peak compression, while the density achieved by the set-up of two intersecting ns laser pulses was slightly lower. A closer observation of the results revealed a finding of major importance about the sustainability of these profiles, since their compression lifetime was limited to a few hundreds of picoseconds, making experimental synchronization with the main accelerating pulse unfeasible, due to the inherent laser system jitter. In contrast, the double intersecting ns laser pulses generated BWs set-up delivered a sufficient compression of ~ 0.55 *n*_*cr*_, sustained for longer than 2.5 ns, as presented in Fig. 3a–d. This set-up led to a peak compression of the initial gas density from approximately 9 × 10^19^ atoms/cm^3^ (0.05 n_cr_) to 10^21^ atoms/cm^3^ i.e., ten times greater. The peak compression density is almost constant for at least 2.5 ns. This important finding secures the experimental synchronization with the main accelerating fs laser pulse and provides the ground for an achievable and efficient experimental implementation. In addition to the aforementioned results, it was shown in our previous work that the intersecting ns laser pulses shaped a cylindrical compression geometry, while the parallel ns laser beams geometry shaped a planar compression in which the density gradients along the Z-direction were absent^36^. Therefore, given the long ~ 2.5 ns compression duration time using the dual pulse intersecting geometry, is considered to be the optimal configuration for experimental implementation.

**Figure 4:**
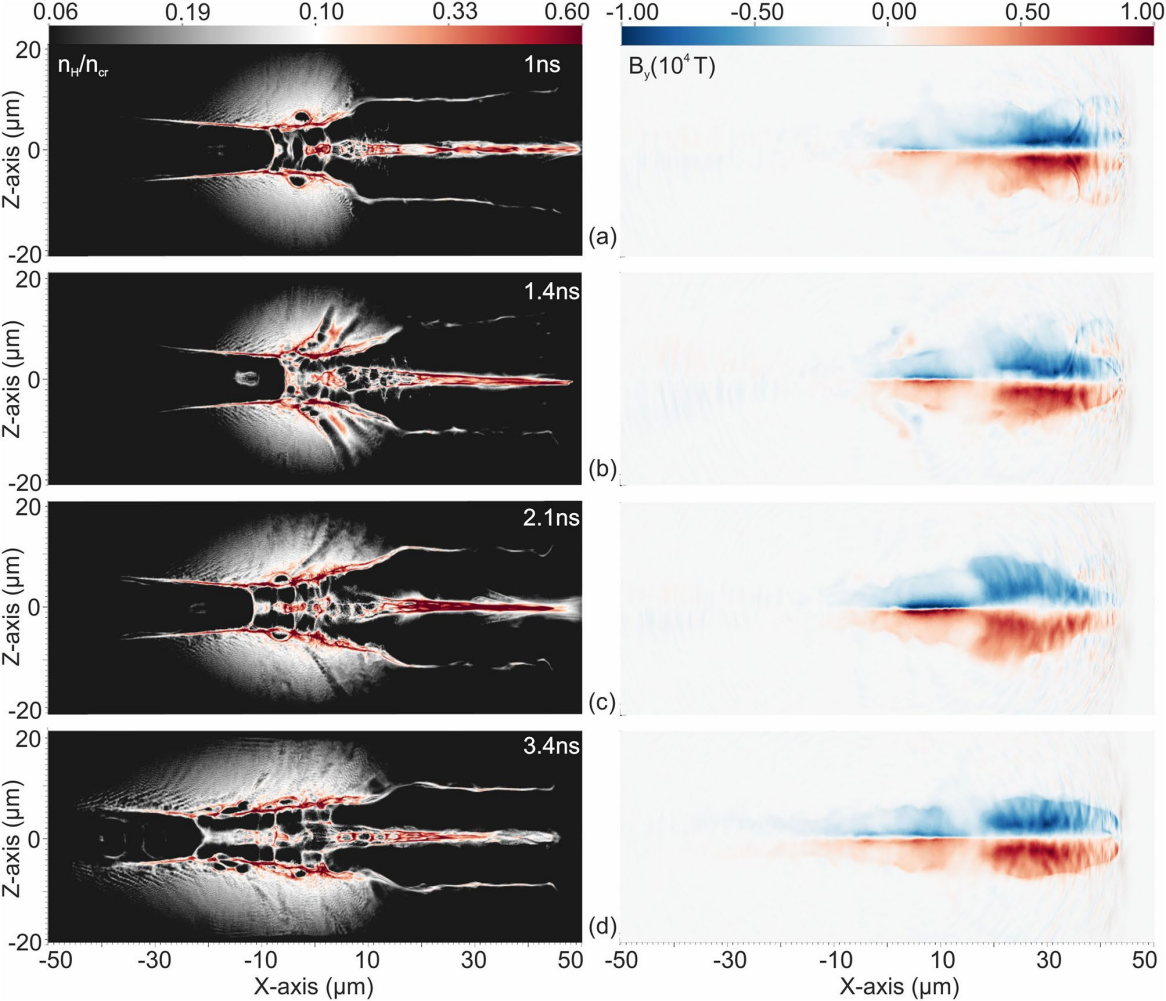
PIC simulation results of the four near critical density target profiles, at 1.0 (**a**), 1.4 (**b**), 2.1 (**c**) and 3.4 ns (**d**), interacting with the 45TW, 23 fs Zeus laser pulse (left) and the corresponding azimuthal magnetic fields.

The efficiency of the energetic proton beam generation by the interaction of the 45 TW, 23 fs Zeus laser pulses with the optically shaped gaseous target generated by this optimal configuration are further studied. PIC models are developed to simulate the proton acceleration of such pulses interacting with the aforementioned optically shaped gaseous target profiles. Figure 3a–d presents a time series of optically shaped density profiles within the ~ 2.5 ns of compression duration generated by the two intersecting ns laser pulses, specifically at 1, 1.4, 2.1 and 3.4 ns respectively. The gaseous target density profiles computed by the MHD simulations at each of these time moments, were then implemented by the PIC models, considering the density gradient along the X- and Z-axis, while the density gradient along the Y-axis was negligible in the interaction area of the intersection laser beams, as presented in Fig. 3e. The initial density distribution of the cylindrically shaped target profile, at 2.1 ns that the maximum compression is achieved, is illustrated in Fig. 3b and the density lineouts, including the top-hat reference profile, are shown in Fig. 3c. The simulation results of the proton densities and the azimuthal magnetic vortex fields, for the four PIC models, are shown in Fig. 4a–d left and right respectively. The resulting proton densities demonstrate the formation of accelerated proton filaments, for each target profile, 600 fs after the interaction. The corresponding azimuthal magnetic vortex magnetic field (B_y_) is greater than 2 × 10^4^ T at 350 fs, for all gas-target profiles. The plasma-vacuum interface usually considered in PIC simulations for top-hat profiles was not considered here, since the realistic profiles were taken into consideration by our PIC models. Consequently, when the fs interaction pulse exits the maximum density area of the target, propagates through the low density plasma, as shown in Fig. 3f at X = 0 μm where the down ramp starts. Thus, the magnetic vortex does not extend transversely, but rather moves along the plasma gradient and expands longitudinally along the length of the plasma waveguide^46^.

**Figure 5:**
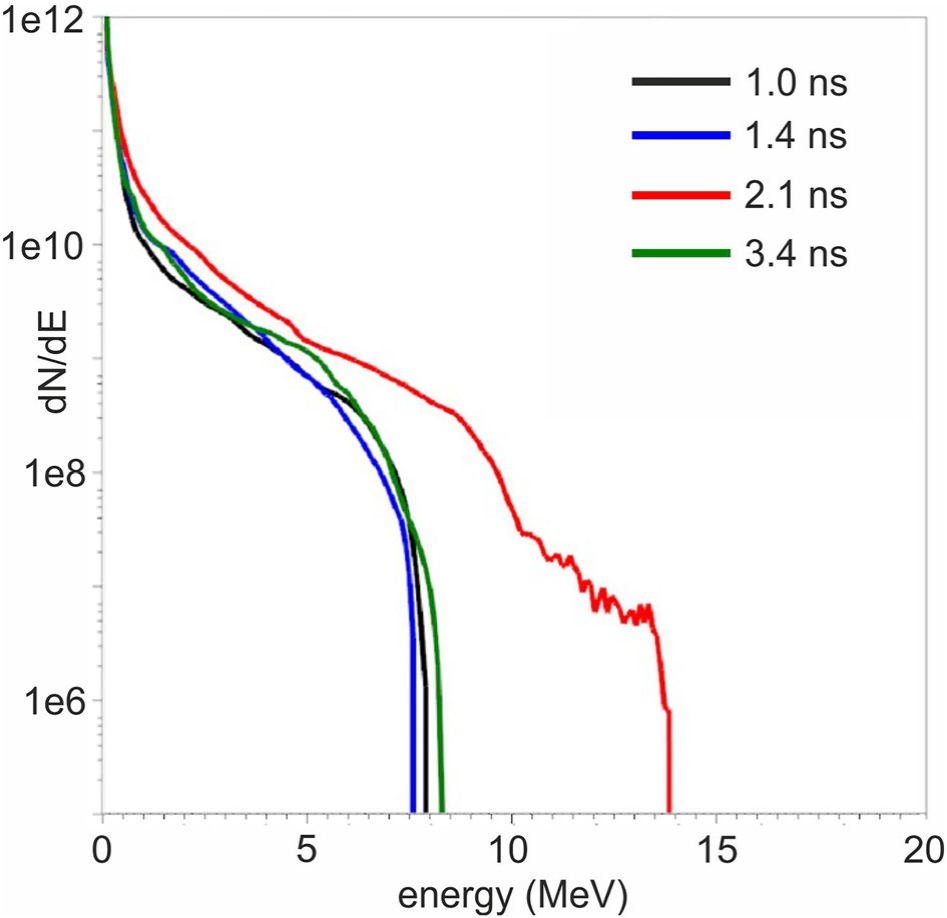
PIC simulation results of the proton energy spectra of the four n_cr_ profile models at 600 fs.

**Figure 6:**
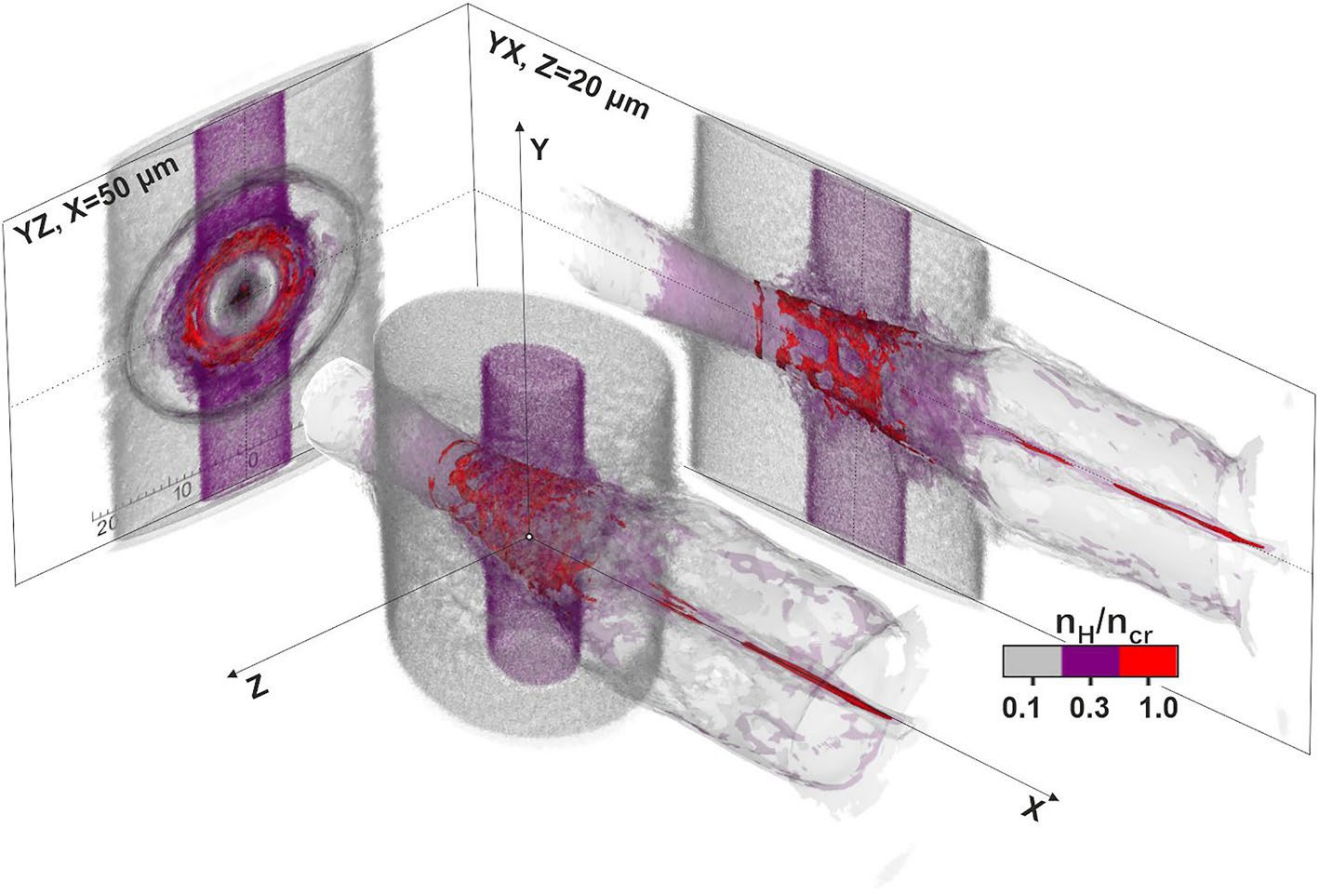
3D iso-surface contour plot snapshot at 600 fs, of the interaction of the main laser pulse with the cylindrical near critical density profile, at 2.1 ns compression time.

Figure 5 shows the proton energy spectra of the four PIC model simulations, indicating the characteristic values of the maximum proton energies achieved. Furthermore, the elongated magnetic vortex field acts as a collimator on the particle beam, making the proton sources to converge to a tightly focused beam. A result of high importance is that all models result to the generation of high energy accelerated protons, suggesting that the compressed gas may serve as a near critical plasma target for proton acceleration whenever the interaction occurs, within the ~ 2.5 ns compression time. The resulting cut-off kinetic proton energies range from 8 to 14 MeV. The maximum proton energy scales with the laser power as $\sim {P}^{0.735}$, in agreement with the laser power scaling law^20^. Furthermore, the interaction leads to accelerated electrons well beyond the ponderomotive scaling^47^, with the most energetic exceeding 60 MeV. Figure 6 shows the 3D iso-surface contour plot of the interaction of the fs main pulse with the cylindrical near critical density profile at 2.1 ns. At 600 fs after the interaction, the PIC simulation results indicate that the protons have gained their maximum kinetic energy and a highly collimated proton beam is formed.

**Figure 7:**
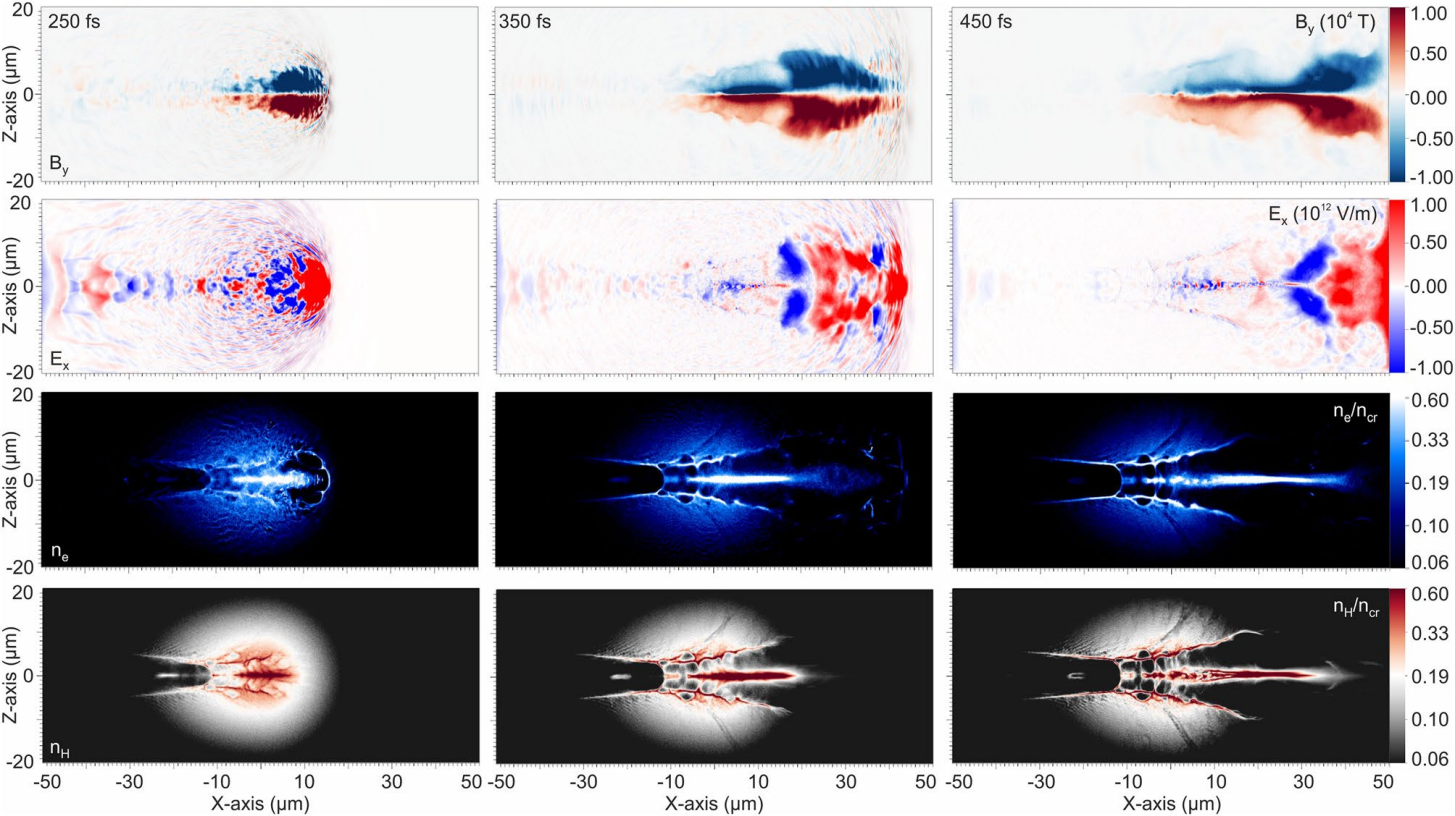
The azimuthal magnetic field B_y_ of the vortex (first row), the longitudinal accelerating electric field E_x_ (second row), the electron and the proton density components (third and fourth rows respectively) in logarithmic scale at t = 250, 350 and 450 fs.

Figure 7 shows the evolution of the azimuthal magnetic field (B_y_), the longitudinal electric field (E_x_) and the proton and electron densities at 250, 350 and 450 fs respectively. At 225 fs the fs interaction laser pulse penetrates the area of the maximum plasma density. The colour-bar for the B_y_ is saturated at 2 × 10^4^ T. The maximum value of the magnetic vortex within the plasma channel exceeds the value of 5 × 10^4^ T and the maximum value of E_x_ is approximately 5 × 10^12^ V/m, at 250 fs. In contradiction to the ideal but unrealistic top-hat plasma case, the expansion of the magnetic vortex within the inhomogeneous plasma with a density gradient expanding forward and lateral, as presented in Fig. 7, leads to the weakening of its azimuthal magnetic field as it expands^21^. The simulation results of the accelerated electrons and protons presented in Fig. 7 demonstrate their bunches, characterized by a high degree of collimation throughout during their acceleration. The proton bunch exhibits a radius of approximately 2 μm at 1/e of its maximum density, 40 μm after the peak density of the target. The laser pulse energy coupled to the plasma was found to be above 48%, indicating a high level of efficiency compared to laser-solid interactions.

## Conclusions and prospects

In this work, the feasibility of proton acceleration by TW, femtosecond laser pulses interacting with high pressure, optically shaped gas-jet profiles were investigated. The optical shaping of the gas-jet profiles via the generation of multiple, counterpropagating Sedov type colliding BWs was investigated performing MHD simulations^36^. The gas-jet profiles were compressed upon the BWs shock front collision, into steep density gradient, short scale length, near critical density target profiles. An intersecting ns laser double pulse layout is identified to be the preferable candidate for the generation of cylindrically compressed target profiles, able to sustain their density distribution for several nanoseconds. These profiles are characterized by a controllable and sustainable compression time window, able to provide plasma density profiles for effective laser induced ion acceleration experiments. The models are developed in 3D, enabling the PIC simulations to achieve the maximum accuracy by considering realistic optically shaped gas target profiles and overcoming the 2D PIC near critical density plasma modelling limitations, which tend to overestimate the computed particle energies^25,48^. The proposed laser pulses layout eliminates the experimental laser-target synchronization restrictions and can make the synchronisation between the interaction fs laser with the optically shaped target experimentally feasible. Tens of MeV’s of protons are accelerated by the 45 TW Zeus fs laser pulses when they interact with these optically shaped target profiles within their nanosecond living compression time window. The simulation results were validated by preliminary experiments using optical probing methods, indicating that for the case of a high pressure Helium gas jet the compression at near critical plasma density was sustained for ~ 4.5 ns which, as expected, is twice as long compared to the numerically predicted compression time for Hydrogen^49^. The experimental campaign for the synchronisation and implementation of these shaped targets in Zeus laser acceleration experiments is in progress.

## Methods

To model the colliding BWs in hydrogen (H) gas-jet targets, the modular, parallel, multiphysics simulation code FLASH^50^ was used. The developed MHD model is based on a customized version of the 3D LaserSlab model^36^. For the generation of the BWs, a 1064 nm wavelength, 835 mJ energy laser pulse of 6 ns duration at the 1/e of the maximum intensity, with a Gaussian spatial profile and a trapezoidal temporal profile and a focal spot of 10 μm diameter was used. This pulse delivers peak intensity *I* = 8.5 × 10^13^ W/cm^2^. A 100 mm focusing lens was considered for tight focusing. The main pulse was split to generate two up to four pulses of equal energy for the generation of the dual to quadruple BWs. The ray tracing module was introduced in the simulations, and each laser pulse was modelled by 2.5 × 10^4^ rays. The model included the opacity tabulated equation-of-state (EOS) IONMIX4, defined over a temperature-density numerical grid, to calculate the absorption, the emission, and the transmission of the laser pulse throughout the H gas target. The gas target interaction with the intense laser pulse was described by the atomic H EOS with a specific heat ratio *γ* = 5/3. The gas density profile was modelled as 3D Gaussian with $n={n}_{0}{\text{exp}}(-\frac{1}{300 \times {10}^{-6}}\left({x}^{2}+0.2{y}^{2}+{z}^{2}\right))$, to decay at a lower rate in the Y-direction where the gas-jet propagates. The H gas was initially 5% ionized, while the degree of ionization was calculated during the simulation, using the H tabulated EOS, as a function of the H temperature and density. The 5% degree of ionization was introduced to the model as an initial condition, to model the artificial percentage adopted for the laser rays to heat the gas while penetrating it^50^. The internal energy and the pressure were also computed by the H tabulated EOS. A gas-jet peak density of 8.7 × 10^19^ cm^−3^ corresponding to the 5% *n*_cr_ for *λ* = 805 nm, was used. The simulation run time was 6 ns, having an initial time step of 100 attoseconds and a Courant–Friedrichs–Lewy (CFL) number of 0.4. A 3D computational domain of (X × Y × Z) = (800 × 600 × 800) μm with sides discretized to 16 cells upon initialization, was developed. The adaptive mesh refinement (AMR) was set to the maximum level of 5, and generated 512 nested cells per axis, resulting to a cell size of ~ (1.5 × 1.2 × 1.5) μm. The hydro and conductivity boundary conditions were set to outflow, allowing shocks to propagate freely out of the domain boundaries. The hybrid Riemann hydrodynamic solver of 2^nd^ order interpolation was used. The simulations of the developed models were performed on the THIN node ‘island’ of the high-performance computer (HPC) of the Advanced Research Information System (ARIS) of the Greek National Infrastructures for Research and Technology (GRNET) on 20 nodes, with 20 CPU cores and 56 GB memory per node. The simulation runtimes of each model were approximately 7 wall-clock hours^51^.

The interaction of the Zeus laser pulse with the steep gradient, near-critical density target profiles was simulated by the 3D PIC code EPOCH^52^. The standard PIC method was implemented, using the Boris pusher and the Yee solver and the current density was computed by the Villasenor and Buneman scheme^53–55^. A three-dimensional domain of (X × Y × Z) = (100 × 40 × 40) μm was discretized by (2000 × 400 × 400) cells to result in a cell size able to resolve the plasma Debye length^19–25,48,56,57^. An initially unionized H gas was considered, following the results obtained by the MHD simulations^36^. The field ionization module of EPOCH was switched on and the H ionization energy was set to be 13.598 eV. Up to 4 macroparticle per cell of 5th particle shape function order were introduced for the simulations to converge and the current smoothing function was adopted to minimize the possible noise and self-heating^52^. The density profile, were the optically shaped gas-jet targets, compressed by colliding BWs generated by nanosecond laser pulses in parallel or intersecting geometries^36^, while a top-hat H density distribution was initially modelled as a reference. The distribution of the density profiles was set to be $n\propto exp(x/{l}_{s})$, where ${l}_{s}$ is the density scale length (1/e of the peak density) to follow the FLASH MHD simulation results of peak densities in the range of 0.16 n_cr_ to 1.1 n_cr_^35,36^. The electron and ion temperatures were set at *T*_*e*_ = *T*_*i*_ = 1.5 eV according to the MHD results within the BWs collision region. The laser pulse was modelled as a linearly polarized Gaussian beam focusing on a spot of 3 μm diameter at Full Width Half Maximum (FWHM), corresponding to an intensity of *I* = 4.5 10^20^ W/cm^2^, with a normalized vector potential *a*_*0*_ = 14.5. The laser pulse was polarized in the Y-direction and propagated in the X-direction. A parametric study was performed to identify the focusing position delivering the optimal scale length of the H density profile. The Rayleigh length of the pulse was *Z*_*R*_ = 25.3 μm. The boundary conditions of the computational domain were set to be open, avoiding particle and/or field reflections on the boundaries. The simulation time was set to 800 fs with a constant timestep of 129.4 attoseconds. The laser intensity regime of the Zeus laser is high above the intensities where processes like binary collisions occur. Additionally, pair production and classical radiation reactions are effective at much higher intensities. Thus, these processes were ignored in our simulations, since their inclusion would further increase the runtime by orders of magnitude, without affecting the simulation results^58^. The high computational demanding simulations of the 3D PIC models described were performed at the HPC ARIS THIN node ‘island’, reserving 30 nodes. The simulation runtimes of our models ranged from 28 to 48 wall-clock hours.

## Data Availability

The datasets used and/or analysed during the current study available from the corresponding author on reasonable request.
